# Supplementary material for: Complexome profiling of the Chlamydomonas psb28 mutant reveals TEF5 as an early PSII assembly factor
Source: Plant Cell. 2025 Mar 17;37(6):koaf055. doi: 10.1093/plcell/koaf055 (PMC12164590; doi:10.1093/plcell/koaf055)
Supplement: koaf055_Supplementary_Data [file koaf055_supplementary_data.zip › psb28_interactive.zip!/plots/intensity/qualityPlots/peptides/CYB4_Cre13.g586550.t1.2.html]

### Cre13.g586550.t1.2

Trivial name: CYB4  
SeqCoverage [median]: 26.80  
Mass: 15.45 kDa  
  
 

| Sequence | MissedCleavage | Mass [Da] | wtIntensity | muIntensity | Score |
| --- | --- | --- | --- | --- | --- |
| EFGVHTVEEVAK | 0 | 1343.67 | 0.00 | 3810.10 | 71.08 |
| HNNRDDAWIIVQHK | 1 | 1744.88 | 0.00 | 1442.37 | 40.07 |
| LAAGEEAAFQK | 0 | 1133.57 | 435.53 | 3046.49 | 128.38 |
|  |  |  | 435.5 | 8299.0 |  |
